# Supplementary material for: Humanizing a CD28 signaling domain affects CD8 activation, exhaustion and stem-like precursors
Source: bioRxiv. 2025 Mar 13:2025.03.10.642460. Preprint. [Version 1] doi: 10.1101/2025.03.10.642460 (PMC11952375; doi:10.1101/2025.03.10.642460)
Supplement: Supplement 1 [file NIHPP2025.03.10.642460v1-supplement-1.pdf]

bioRxiv preprint doi: https://doi.org/10.1101/2025.03.10.642460; this version posted March 13, 2025. The copyright holder for this preprint (which was not certified by peer review) is the author/funder, who has granted bioRxiv a license to display the preprint in perpetuity. It is made available under aCC-BY 4.0 International license.

List of Supplementary Materials:

Fig S1) Enhanced CD28 signaling in CD28A210P mice does not alter T cell development or homeostatic peripheral T cell populations

Fig S2) CD28^{A210P} mice undergo a proinflammatory response to CD28 superagonist antibody extended

Fig S3) Enhancing CD28 signaling increases the initial effector CD8^{+} T cell response to acute and chronic infection extended

Fig S4) Enhancing CD28 C-terminal signaling induces early upregulation of CD8^{+} T cell inhibitory receptors and expedites exhaustion extended

Fig S5) CD28^{A210P} T cells display enhanced JunB nuclear localization compared to WT while other CD28-dependent signaling is similar to WT T cells

Fig S6) Enhancing CD28 increases Tpex differentiation and long-term maintenance without compromising memory precursor extended

bioRxiv preprint doi: https://doi.org/10.1101/2025.03.10.642460; this version posted March 13, 2025. The copyright holder for this preprint (which was not certified by peer review) is the author/funder, who has granted bioRxiv a license to display the preprint in perpetuity. It is made available under aCC-BY 4.0 International license.

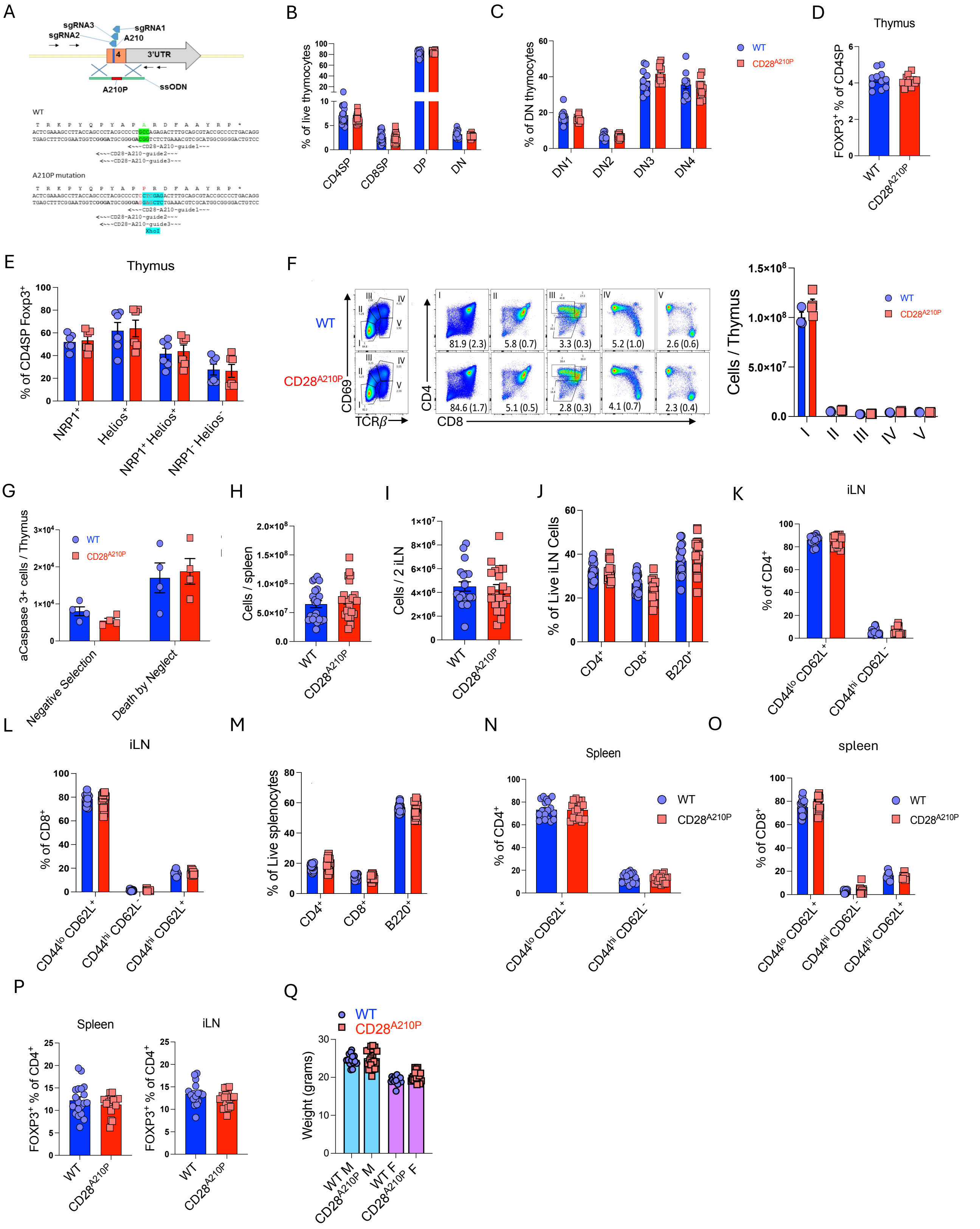

Fig S1) Enhanced CD28 signaling in CD28A210P mice does not alter T cell development or homeostatic peripheral T cell populations
A) Target DNA sequence of CRISPR/Cas9 sgRNAs. In blue: sequence for XhoI enzyme digestion to facilitate genotyping of CD28A210P mice. **B-G)** Flow cytometry analysis of adult thymocytes. **B)** Frequencies of thymocyte subpopulations **C)** Frequencies of DN subpopulations among the DN cells, Gated using CD44 and CD25. **D)** Frequencies of Foxp3^{+} among CD4SP thymocytes. **E)** Frequencies of NRP1^{+} and Helios^{+} cells among thymic Foxp3^{+} Tregs. **F)** Representative gating and absolute counts of thymic developmental subsets. **G)** Positively and negatively selected cells based on cleaved caspase-3 staining in signaled and not signaled thymocytes determined by CD5 and TCR-β. **H-P)** Flow cytometry analysis of adult spleens and inguinal lymph nodes. **H)** Absolute counts of splenocytes. **I)** Absolute counts of inguinal lymph node cells. **J)** Frequencies of CD4^{+}, CD8^{+}, and B220^{+} cells among live inguinal lymph node cells. **K)** Frequencies of CD44^{hi} CD62L^{-} and CD44^{lo} CD62L^{+} CD4^{+} cells in inguinal lymph nodes. **L)** Frequencies of CD44^{lo} CD62L^{+}, CD44^{hi} CD62L^{-}, and CD44^{hi} CD62L^{+} CD8^{+} splenocytes. **M)** Frequencies of CD44^{lo} CD62L^{+}, CD44^{hi} CD62L^{-}, and CD44^{hi} CD62L^{+} CD8^{+} cells in inguinal lymph nodes. **N)** Frequencies of CD4^{+}, CD8^{+}, and B220^{+} cells among live splenocytes. **O)** Frequencies of CD44^{hi} CD62L^{-} and CD44^{lo} CD62L^{+} CD4^{+} splenocytes. **P)** Frequencies of Foxp3^{+} of CD4^{+} cells in spleens and inguinal lymph nodes. **Q)** Body weights of adult male and female WT and CD28^{A210P} mice. Data are pooled or representative of at least two experiments with significance assessed by Student's t-test or one-way ANOVA, * = p < 0.05, ** = p < 0.01, *** = p < 0.001.

bioRxiv preprint doi: https://doi.org/10.1101/2025.03.10.642460; this version posted March 13, 2025. The copyright holder for this preprint (which was not certified by peer review) is the author/funder, who has granted bioRxiv a license to display the preprint in perpetuity. It is made available under aCC-BY 4.0 International license.

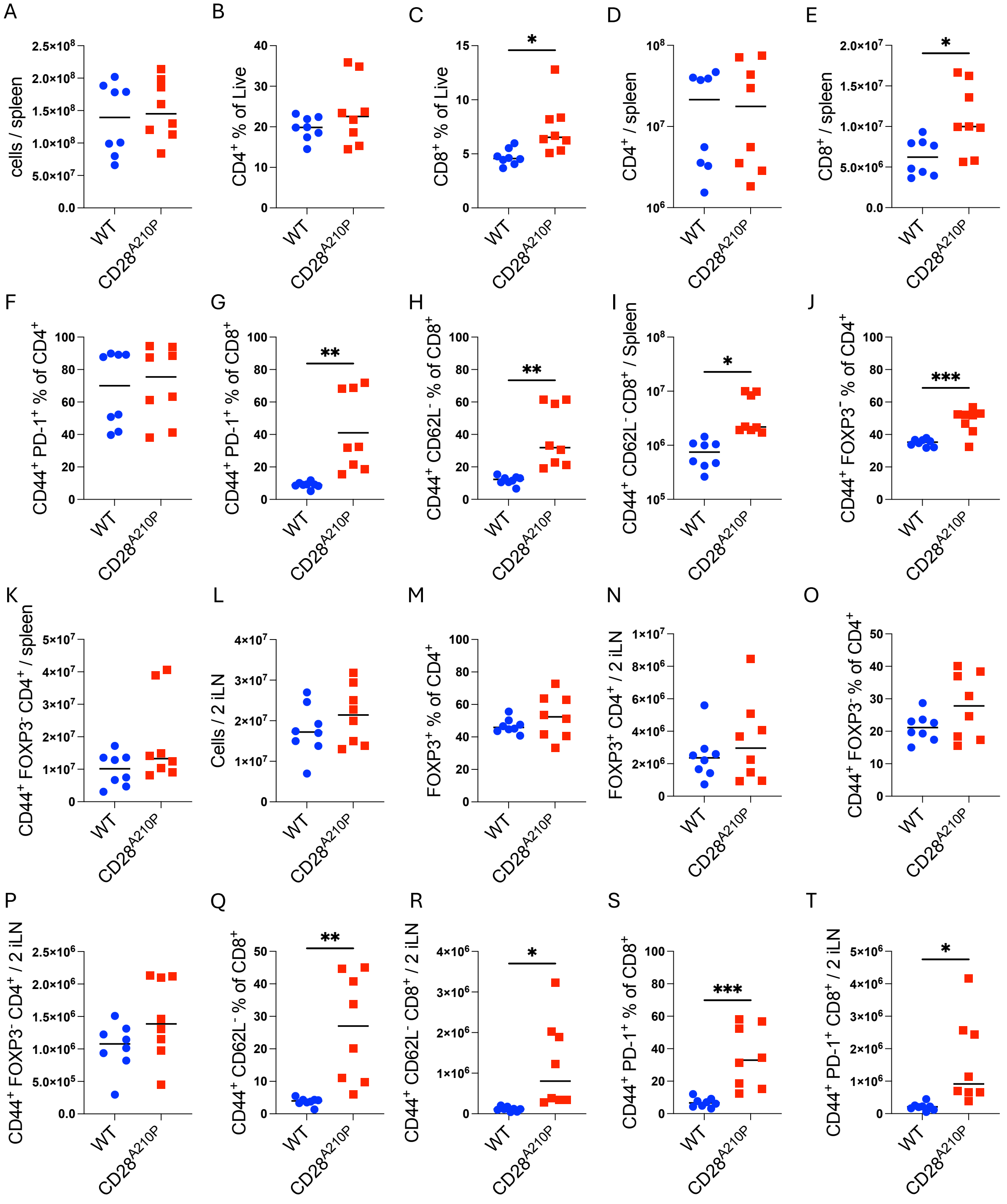

Fig S2) CD28^{A210P} mice undergo a proinflammatory response to CD28 superagonist antibody extended
A-K) Flow cytometric analysis of splenocytes from WT and CD28A210P mice 4 days post injection with CD28 superagonist antibody. Frequencies shown as % of parent population and absolute numbers calculated from total tissue cell counts. **L-T)** Flow cytometric analysis of cell from pooled 2 inguinal lymph nodes of WT and CD28A210P mice 4 days post injection with CD28 superagonist antibody. Frequencies shown as % of parent population and absolute numbers calculated from total tissue cell counts. Data are pooled from two independent experiments with significance assessed by Student's t-test, * = $p < 0.05$, ** = $p < 0.01$, *** = $p < 0.001$.

bioRxiv preprint doi: https://doi.org/10.1101/2025.03.10.642460; this version posted March 13, 2025. The copyright holder for this preprint (which was not certified by peer review) is the author/funder, who has granted bioRxiv a license to display the preprint in perpetuity. It is made available under aCC-BY 4.0 International license.

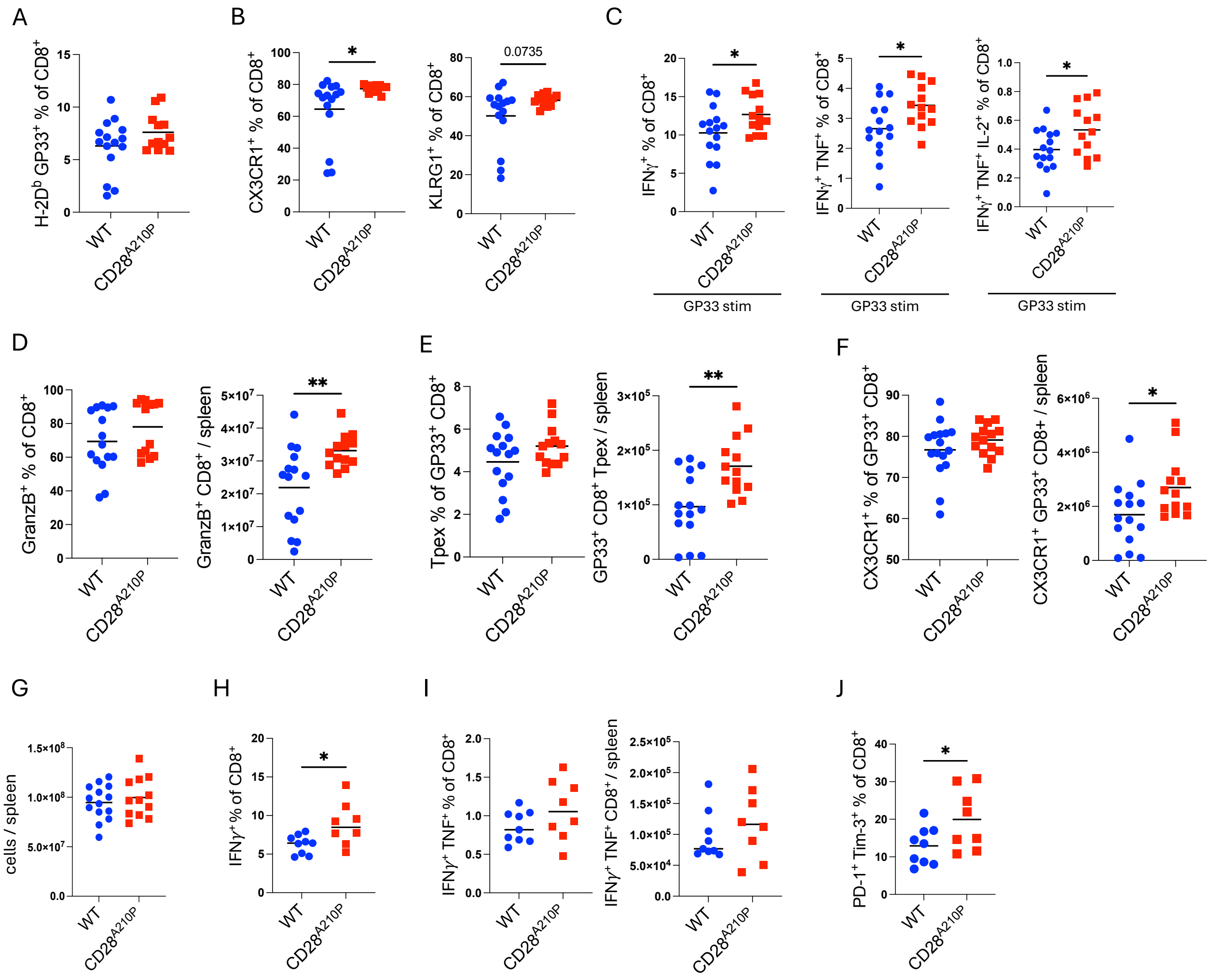

Fig S3) Enhancing CD28 signaling increases the initial effector $CD8^+$ T cell response to acute and chronic infection extended A-F) Flow cytometry data from WT and CD28A210P splenocytes 7 days post infection with LCMV Armstrong. For intracellular cytokine staining (C,D) splenocytes were restimulated ex vivo for 4 hours with GP33 peptide in the presence of GolgiPlug. GP33 tetramer staining were done for 1 hour at 4C. Tpex = $CD8^+$ $PD\text{-}1^+$ $SLAMF6^+$ $Tim\text{-}3^-$. $GP33^+$ = H-$2D^b$ GP33 $tetramer^+$. **G-J)** Flow cytometry data from WT and $CD28^{A210P}$ splenocytes 8 days post infection with LCMV clone 13. For intracellular cytokine staining (H,I) splenocytes were restimulated ex vivo for 4 hours with GP33 peptide in the presence of GolgiPlug. J) $PD\text{-}1^+$ $Tim\text{-}3^+$ frequencies among $CD8^+$ cells. Data are pooled from or representative of two independent experiments with significance assessed by Student's t-test, * = $p < 0.05$, ** = $p < 0.01$, *** = $p < 0.001$.

bioRxiv preprint doi: https://doi.org/10.1101/2025.03.10.642460; this version posted March 13, 2025. The copyright holder for this preprint (which was not certified by peer review) is the author/funder, who has granted bioRxiv a license to display the preprint in perpetuity. It is made available under aCC-BY 4.0 International license.

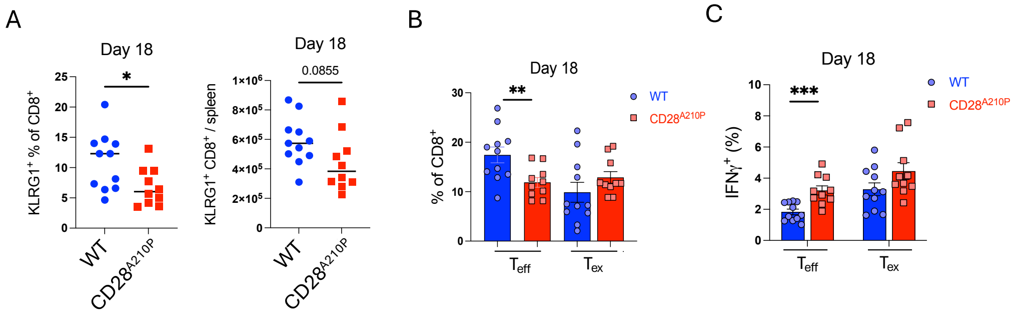

Fig S4) Enhancing CD28 C-terminal signaling induces early upregulation of CD8⁺ T cell inhibitory receptors and expedites exhaustion extended A-C) Flow cytometry analysis of spleens from WT and CD28^{A210P} mice infected 18 days prior with LCMV clone 13. **A)** Frequencies and absolute numbers of KLRG1^{+} CD8^{+} cells. **B)** Frequencies of CD8^{+} effector and exhausted cells. **C)** Analysis of intracellular cytokine production of D18 LCMV clone 13 infected WT and CD28^{A210P} splenocytes restimulated ex vivo with GP33 peptide for 4 hours in the presence of GolgiPlug. Effector = CD8^{+} CX3CR1^{+} Exhausted = CD8^{+} CX3CR1^{-} PD-1^{+} Tim-3^{+}. Data are pooled from two independent experiments with significance assessed by Student's t-test, * = $p < 0.05$, ** = $p < 0.01$, *** = $p < 0.001$.

bioRxiv preprint doi: https://doi.org/10.1101/2025.03.10.642460; this version posted March 13, 2025. The copyright holder for this preprint (which was not certified by peer review) is the author/funder, who has granted bioRxiv a license to display the preprint in perpetuity. It is made available under aCC-BY 4.0 International license.

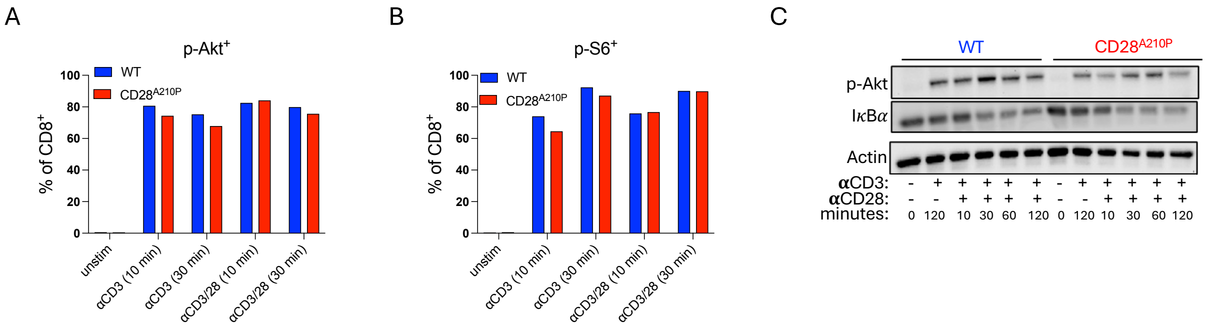

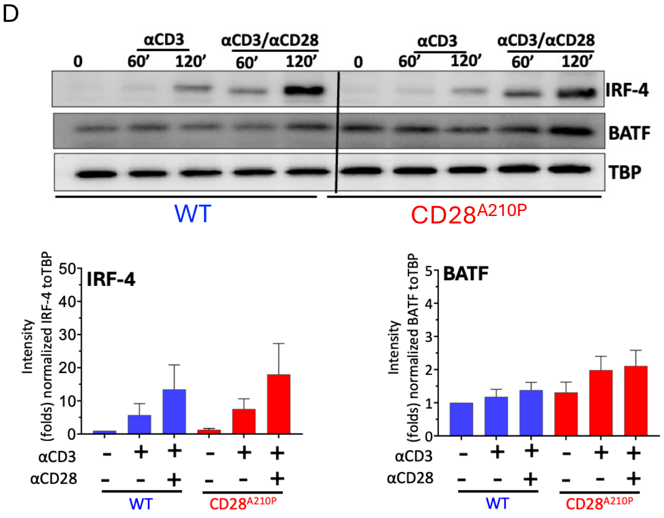

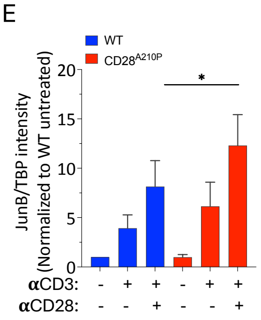

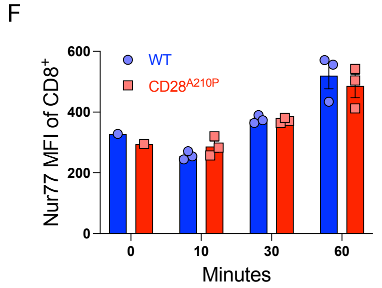

Fig S5) CD28^{A210P} T cells display enhanced JunB nuclear localization compared to WT while other CD28-dependent signaling is similar to WT T cells A-B) WT or CD28^{A210P} CD8$^+$ T cells were enriched from secondary lymphoid organs using MACS enrichment. Cells were then stimulated with plate bound agonistic anti-CD3/28 (1 μg/mL). Cells were stimulated for 10-30 minutes as indicated and then harvested for p-flow cytometric analysis of p-Akt and p-S6. Data representative of 3 independent experiments. **C-D)** WT or CD28^{A210P} CD8$^+$ T cells were stimulated with plate bound agonistic anti-CD3/28 (5 μg/mL) as indicated. Cells were stimulated for up to 2 hours prior to lysis for immunoblot analysis. Akt phosphorylation (S473) and IKBα degradation assessed from cytoplasmic fractions and IRF4 and BATF assessed from nuclear extracts. C) is representative of 2 independent experiments and D) is representative of 3-4 independent experiments. **E)** WT or CD28^{A210P} CD8$^+$ T cells were stimulated with plate bound agonistic anti-CD3/28 (5 μg/mL) as indicated. Cells were stimulated for 2 hours prior to nuclear extraction for immunoblot analysis, quantification of 5 pooled experiments shown. **F)** WT or CD28^{A210P} CD8$^+$ T cells were stimulated with plate bound agonistic anti-CD3/28 (5 μg/mL) for indicated times. Nur77 staining among CD8$^+$ cells was quantified. Data are pooled or representative of 3-4 independent experiments with significance assessed by Student's t-test or one-way ANOVA , * = $p < 0.05$, ** = $p < 0.01$, *** = $p < 0.001$.

bioRxiv preprint doi: https://doi.org/10.1101/2025.03.10.642460; this version posted March 13, 2025. The copyright holder for this preprint (which was not certified by peer review) is the author/funder, who has granted bioRxiv a license to display the preprint in perpetuity. It is made available under aCC-BY 4.0 International license.

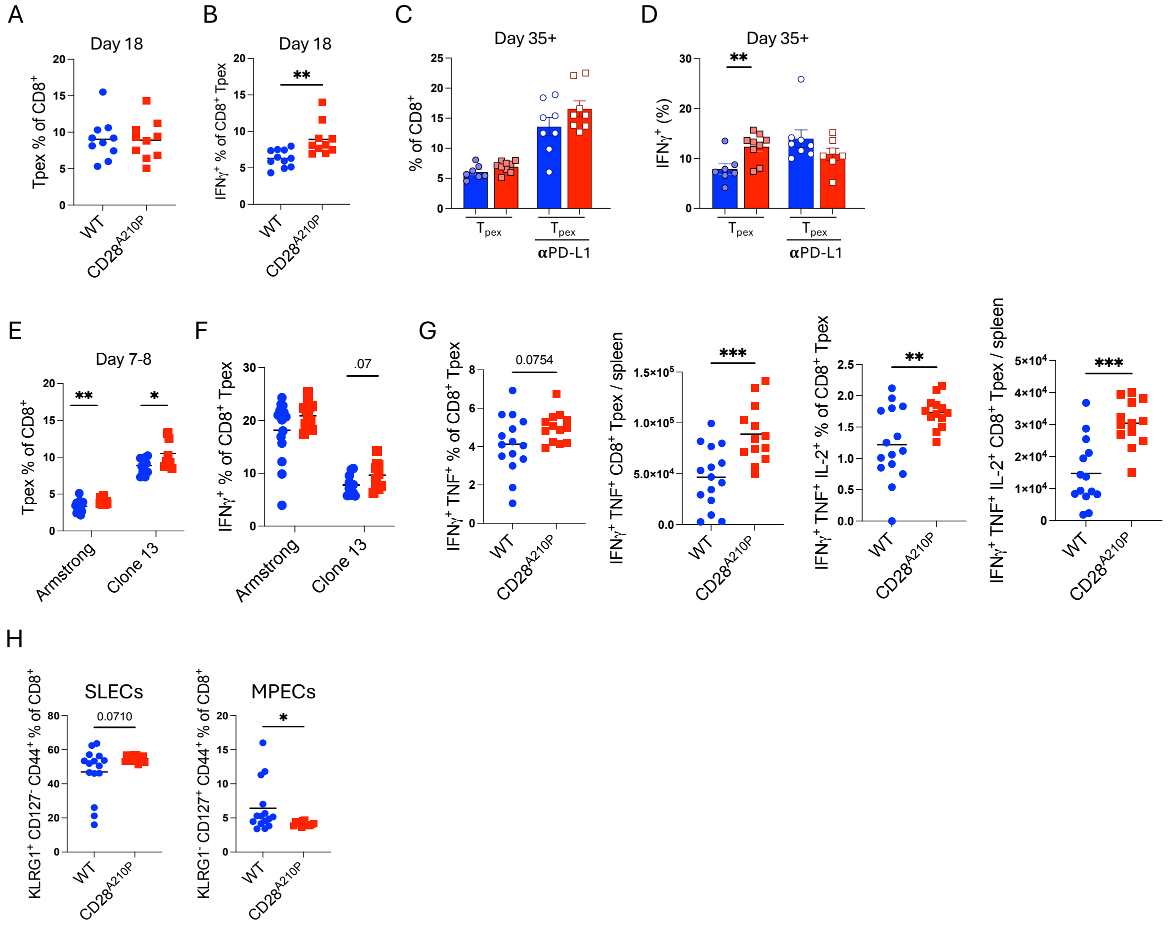

Fig S6) Enhancing CD28 increases Tpex differentiation and long-term maintenance without compromising memory precursor extended

A-B) WT or CD28^{A210P} mice were infected with LCMV Clone 13. 18 days post infection splenocytes were stained for flow cytometric analysis. **A)** Frequencies of CD8$^+$ Tpex. **B)** Intracellular cytokine staining of splenocytes restimulated with LCMV GP33 peptide ex vivo for 4 hours in the presence of GolgiPlug. **C-D)** WT or CD28^{A210P} mice were infected with LCMV Clone 13 and 35+ days post infection splenocytes were stained for flow cytometric analysis. For mice treated with anti-PD-L1 (10F.9G2), 5 injections of anti-PD-L1 were administered I.P. (200 μg/injection) every 3 days for 2 weeks prior to analysis. **C)** Frequencies of Tpex among CD8$^+$ cells. **D)** Frequencies of IFNγ^+ cells following ex vivo GP33 peptide restimulation. **E-F)** Flow cytometric analysis of spleens from LCMV Armstrong or clone 13 infected WT and CD28^{A210P} mice (D7-8). **E)** Frequencies of Tpex among CD8$^+$ cells. **F)** Frequencies of IFNγ^+ cells following ex vivo GP33 peptide restimulation. **G-H)** Flow cytometry of spleens from LCMV Armstrong infected WT and CD28^{A210P} mice (Day 7). **G)** Frequencies and absolute counts of cytokine producing CD8$^+$ Tpex following ex vivo GP33 peptide restimulation. **H)** Frequencies of SLECs and MPECs among CD8$^+$ cells. For Tpex = CD8$^+$ PD-1$^+$ SLAMF6$^+$ Tim-3$^-$. Data are pooled from 2 independent experiments with significance assessed by Student's t-test, * = $p < 0.05$, ** = $p < 0.01$, *** = $p < 0.001$.
